# Digestible and metabolizable energy in soybean meal and soybean hulls when fed to growing pigs or sows

**DOI:** 10.1093/tas/txaf041

**Published:** 2025-03-27

**Authors:** Yeonwoo Kim, Su A Lee, Hans H Stein

**Affiliations:** Division of Nutritional Sciences, University of Illinois at Urbana-Champaign, Urbana, IL, 61801, USA; Department of Animal Sciences, University of Illinois at Urbana-Champaign, Urbana, IL, 61801, USA; Division of Nutritional Sciences, University of Illinois at Urbana-Champaign, Urbana, IL, 61801, USA; Department of Animal Sciences, University of Illinois at Urbana-Champaign, Urbana, IL, 61801, USA

**Keywords:** digestibility, energy, sows, soybean hulls, soybean meal

## Abstract

An experiment was conducted to test the hypothesis that the apparent total tract digestibility (**ATTD**) of gross energy (**GE**) and concentrations of digestible energy (**DE**) in soybean meal (**SBM**) and soybean hulls are greater when fed to gestating sows or lactating sows than to growing pigs, and that there is no difference in ATTD of GE between gestating and lactating sows. Three experimental diets were prepared. The basal diet consisted of corn as the sole source of energy, and two additional diets contained corn and 30% SBM or corn and 20% soybean hulls. All diets were fed to growing pigs and gestating and lactating sows. Twenty-four growing pigs and twenty-four gestating sows were housed in metabolism crates, and fecal and urine samples were quantitatively collected. Twenty-four lactating sows were housed in farrowing crates and feces were grab-sampled. The ATTD of GE, DE, and metabolizable energy (**ME**) were calculated in diets fed to growing pigs and gestating sows, and DE and ME in SBM and soybean hulls were calculated as well. The ATTD of GE and DE were also determined in diets fed to lactating sows, and DE was determined for SBM and soybean hulls. Results from growing pigs indicated that DE and ME were greater (*P* < 0.05) in corn and SBM compared with soybean hulls. For gestating sows, DE in corn and SBM was also greater (*P* < 0.05) than in soybean hulls, and ME in corn was greater (*P* < 0.05) than in SBM, whereas soybean hulls had the least (*P* < 0.05) ME. Results for lactating sows indicated that DE in corn and SBM was greater (*P* < 0.05) than in soybean hulls, but lactating sows had greater (*P* < 0.05) DE for soybean hulls than gestating sows and growign pigs, whereas gestating sows had greater (*P* < 0.05) DE for corn than lactating sows. Gestating sows also had greater (*P* < 0.05) ME for corn than growing pigs whereas growing pigs had greater (*P* < 0.05) ME for SBM than gestating sows. In conclusion, soybean hulls contain less DE and ME than corn and SBM, but there are no consistent differences in DE and ME among growing pigs, gestating, and lactating sows.

## Teaser

Soybean meal and soybean hulls have greater digestible energy when fed to growing pigs or sows than previously thought

## INTRODUCTION

Values for digestible energy **(DE)** and metabolizable energy **(ME)** in feed ingredients are usually determined in growing pigs and subsequently applied to all groups of pigs. Results of recent research, however, indicates that the energy concentration of soybean meal (**SBM**) is greater than previously thought when fed to growing pigs (Sotak-Peper et al., 2015; Lopez et al., 2020; Lee et al., 2021). This increase in energy may be a result of changes in genetic status of pigs or differences in methodologies used to measure DE and ME, but there are no recent experiments assessing DE and ME in SBM when fed to sows. Therefore, it remains unclear if sows also have greater DE and ME in SBM compared with current book values (NRC, 2012).

Due to hindgut fermentation, sows can usually utilize energy in fiber-rich ingredients better than growing pigs (Casas and Stein, 2017), but it is not known if that is also the case for SBM, which is not a high fiber ingredient although SBM contains more fiber than corn (NRC, 2012). Although soybean hulls contain less DE and ME than corn and SBM, the DE and ME in soybean hulls may be greater in sows than in growing pigs because soybean hulls is a high fiber ingredient that is presumed to be better fermented by sows than by growing pigs. However, there are no recent assessments of DE and ME in soybean hulls fed to sows. Likewise, it is not known if feed ingredients have the same DE and ME for lactating sows as gestating sows because most research into energy values of feed ingredients fed to sows has focused on gestating and not lactating sows (Shi and Noblet, 1993; Lowel et al., 2015; Casas and Stein, 2017). However, because lactating sows are usually allowed ad libitum access to their diets, it is uncertain if they have different DE and ME of feed ingredients than gestating sows, and to our knowledge, this hypothesis has not been experimentally tested. Therefore, an experiment was conducted to test the hypothesis that DE and ME of SBM and soybean hulls are greater when fed to gestating or lactating sows than to growing pigs, but that there is no difference in apparent total tract digestibility (**ATTD**) of gross energy (**GE**) and DE between gestating and lactating sows. The second hypothesis was that DE and ME in SBM fed to both growing pigs and sows are greater than current book values and greater than in corn regardless of the physiological state of the animal.

## MATERIALS AND METHODS

All animal procedures were approved by the Institutional Animal Care and Use Committee at the University of Illinois, Urbana, IL, USA, before the experiment was initiated. One batch of SBM was procured from Archer Daniels Midland Corporation (Decatur, IL, USA) and one batch of soybean hulls was procured from South Central FS (Watson, IL, USA). Locally grown corn was obtained from the University of Illinois Feed Mill (Table 1), and this batch was used in all diets. There were three dietary treatments, and all diets were formulated to contain Ca, P, and all micronutrients at or above the requirements for growing pigs, which is also above the requirement for gestating and lactating sows (NRC, 2012). A basal diet contained corn as the sole source of energy, and two additional diets contained corn and 30% SBM or corn and 20% soybean hulls as the energy sources (Table 2). Titanium dioxide was included in all diets to allow for the calculation of digestibility in lactating sows and all pigs were fed the same diets.

**Table 1.** Analyzed nutrient composition of feed ingredients, as-is basis

Item, %	Corn	Soybean meal	Soybean hulls
Dry matter	86.50	87.89	89.32
Gross energy, kcal/kg	3,935	4,110	3,832
Ash	1.42	6.26	4.81
Crude protein	7.18	47.32	11.83
Acid hydrolyzed ether extract	3.08	2.08	1.95
Starch	61.58	-	-
Total dietary fiber	13.4	20.2	71.9
Soluble dietary fiber	2.2	2.9	8.6
Insoluble dietary fiber	11.2	17.3	63.3
Soluble-non-starch polysaccharide			
Rhamnose	ND^1^	0.03	0.30
Fucose	ND	0.04	0.01
Arabinose	0.12	0.15	0.51
Xylose	0.05	ND	ND
Mannose	0.12	0.31	2.06
Galactose	ND	0.31	1.15
Total soluble-non-starch polysaccharide	0.29	0.84	4.03
Insoluble-non-starch polysaccharide			
Rhamnose	ND	0.21	0.37
Fucose	ND	0.29	0.21
Arabinose	1.35	2.12	3.85
Xylose	2.12	1.12	7.38
Mannose	0.15	0.71	3.32
Galactose	0.39	4.40	1.82
Glucose	0.92	0.31	1.74
Cellulose	1.93	3.46	30.99
Total insoluble-non-starch polysaccharide	6.86	12.62	49.68
Raffinose	0.15	0.81	0.10
Stachyose	0.01	3.57	0.81
Indispensable amino acids			
Arginine	0.37	3.20	0.64
Histidine	0.20	1.25	0.31
Isoleucine	0.25	2.37	0.53
Leucine	0.75	3.52	0.84
Lysine	0.29	3.02	0.85
Methionine	0.15	0.62	0.14
Phenylalanine	0.33	2.41	0.51
Threonine	0.25	1.72	0.45
Tryptophan	0.05	0.63	0.06
Valine	0.36	2.35	0.59
Total	3.00	21.09	4.92
Dispensable amino acids			
Alanine	0.50	1.98	0.52
Aspartic acid	0.49	5.32	1.21
Cysteine	0.16	0.69	0.23
Glutamic acid	1.23	8.3	1.56
Glycine	0.31	1.95	0.89
Proline	0.58	2.28	0.65
Serine	0.29	1.97	0.59
Tyrosine	0.20	1.55	0.46
Total	3.76	24.04	6.11
Total amino acids	6.76	45.13	11.03

^1^ND, not detected.

**Table 2.** Ingredient composition of experimental diets^1^, as-fed basis

Item	Basal	Soybean meal	Soybean hulls
Ground corn, %	96.52	66.92	76.74
Soybean meal, %	-	30.00	-
Soybean hulls, %	-	-	20.00
Dicalcium phosphate, %	1.46	1.00	1.60
Ground limestone, %	0.72	0.78	0.36
Titanium dioxide, %	0.40	0.40	0.40
Sodium chloride, %	0.40	0.40	0.40
Vitamin-mineral premix^1^, %	0.50	0.50	0.50
Analyzed composition			
Dry matter, %	86.71	86.66	87.18
Crude protein, %	6.93	18.89	8.65
Ash, %	3.55	4.52	4.25
Gross energy, kcal/kg	3,793	3,849	3,781

^1^The vitamin-micromineral premix provided the following quantities of vitamins and micro minerals per kg of complete diet: vitamin A as retinyl acetate, 10,622 IU; vitamin D_3_ as cholecalciferol, 1,660 IU; vitamin E as _DL_-alpha-tocopherol acetate, 66 IU; vitamin K as menadione nicotinamide bisulfate, 1.40 mg; thiamin as thiamin mononitrate, 1.08 mg; riboflavin, 6.49 mg; pyridoxine as pyridoxine hydrochloride, 0.98 mg; vitamin B_12_, 0.03 mg; _D-_pantothenic acid as _D-_calcium pantothenate, 23.2 mg; niacin, 43.4 mg; folic acid, 1.56 mg; biotin, 0.44 mg; Cu, 20 mg as copper chloride; Fe, 123 mg as iron sulfate; I, 1.24 mg as ethylenediamine dihydroiodide; Mn, 59.4 mg as manganese hydroxychloride; Se, 0.27 mg as sodium selenite and selenium yeast; and Zn, 124.7 mg as zinc hydroxychloride.

### Growing Pigs

Twenty-four growing gilts and barrows (initial body weight: 40.51 ± 2.83 kg) that were the offspring of line 800 males mated to Camborough females (PIC, Hendersonville, TN, USA) were allotted to one of three diets in a randomized complete block design with three diets and eight replicate pigs per diet. Sex was the blocking factor. Pigs were fed three times the maintenance requirement for energy (i.e., 197 × kcal ME per kg body weight^0.60^; NRC, 2012) and had free access to water throughout the experiment. Pigs were fed 1.52 to 1.89 kg of feed per day depending on their weight. Pigs were housed individually in metabolism crates (0.81 m × 1.52 m). A screen and a urine pan were placed under the slatted floor to allow for the total, but separate, collection of urine and fecal materials.

Daily feed allotments were divided into two equal meals that were provided at 0800 and 1600 h. Feed consumption was recorded daily. The initial seven days were considered the adaptation period to the diet, and urine and fecal materials were collected for the following four days according to the marker-to-marker approach (Adeola, 2001). Fecal collection was initiated when the first marker (i.e., chromic oxide) appeared in the feces and ceased when the second marker (i.e., ferric oxide) appeared. Markers were uniformly mixed in the first meal of d 8 and d 12 at 1% inclusion. Urine was collected in urine buckets over a preservative of 50 mL of 3 *N* HCl, which was added to each empty bucket every day after collection. The weights of orts, feces, and urine samples were recorded, and all fecal samples and 10% of the collected urine were stored at -20°C immediately after collection.

### Gestating Sows

Twenty-four gestating Camborough females (Pig Improvement Company, Hendersonville, TN, USA) that were approximately 65 days into gestation (parity two to six) were allotted to two blocks of 12 sows using a randomized complete block design with three diets and four sows per diet in each block for a total of eight replicate sows for each diet. Breeding group was the blocking factor. Experimental diets were identical to those used for growing pigs. Gestating sows were fed at 1.5 times the maintenance energy requirement for gestating sows (i.e., 100 × kcal ME per kg body weight^0.75^; NRC, 2012). Concentrations of ME in diets were calculated based on NRC (2012). Daily feed allotments were provided twice daily at 0700 and 1600 h. Sows were housed individually in metabolism crates (2.10 m × 0.99 m) that were equipped with a self-feeder, a nipple drinker, and a fully slatted T-bar floor. A screen floor and a urine pan were installed under the T-bar floor to allow for the collection of feces and urine. Experimental diets were fed for 13 days. Feces and urine were collected for four days, as detailed above for growing pigs.

### Lactating Sows

Twenty-four multiparous lactating Camborough females (Pig Improvement Company, Hendersonville, TN, USA) were used in a randomized complete block design with two blocks of 12 sows, three diets, and four sows per diet in each block for a total of eight replicate sows per treatment. Breeding group was the blocking factor. The lactating sows used in the experiment were different from the gestating sows used. Sows were moved to farrowing crates seven days before farrowing and remained there until weaning on day 20 post-farrowing. Feeding of experimental diets started on day five post-farrowing. Sows had seven days of adaptation to the diets and fecal samples were collected via rectal palpation for five days starting on day 12 of lactation. Collected fecal samples were immediately stored at -20°C. Lactating sows had ad libitum access to diets and water throughout the experiment.

### Chemical Analysis

At the conclusion of animal feeding, urine samples from growing pigs and gestating sows were thawed and mixed, and a sub-sample was lyophilized before analysis (Kim et al., 2009). Fecal samples from all groups of animals were thawed and dried in a 55°C forced-air drying oven for seven days (Heratherm OMH750; Thermo Fisher 1873 Scientific Inc., Waltham, MA, USA). Samples were then ground through a 1-mm screen using a hammermill (model MM4; Schutte Buffalo, NY, USA), mixed, and sub-sampled for analysis.

Ingredients, diets, and fecal samples were analyzed for dry matter (**DM**; method 930.15; AOAC Int., 2019). Diets and ingredient samples, fecal samples, and lyophilized urine samples from growing pigs and gestating sows were analyzed for GE on an isoperibol bomb calorimeter (Model 6400, Parr Instruments, Moline, IL, USA) using benzoic acid as the internal standard. All diets and ingredients were also analyzed for ash (method 942.05; AOAC Int., 2019), and ingredients were analyzed for insoluble dietary fiber (**IDF**) and soluble dietary fiber (**SDF**) according to method 991.43 (AOAC Int., 2019) using the Ankom^TDF^ Dietary Fiber Analyzer (Ankom Technology, Macedon, NY, USA). Total dietary fiber (**TDF**) was calculated as the sum of IDF and SDF. Nitrogen was analyzed in ingredients and diets by combustion using a LECO FP628 Nitrogen Analyzer (LECO Corp., Saint Joseph, MI, USA; method 990.03; AOAC Int., 2019), and crude protein was calculated as nitrogen × 6.25. Acid hydrolyzed ether extract was analyzed in ingredients by acid hydrolysis using 3 *N* HCl (AnkomHCl, Ankom Technology, Macedon, NY) followed by crude fat extraction using petroleum ether (method 2003.06; AOAC Int., 2019) on an Ankom fat analyzer (AnkomXT15, Ankom Technology, Macedon, NY, USA). Titanium was analyzed in diets and fecal samples from lactating sows (method 985.01 A, B and C; AOAC Int., 2019) using inductively coupled plasma-optical emission spectrometry (ICP-OES; Avio 200, PerkinElmer, Waltham, MA, USA). Sample preparation included dry ashing at 600°C for 4 h (method 942.05; AOAC Int., 2019) and wet digestion with sulfuric acids (method 3050 B; U.S. Environmental Protection Agency, 2000). Ingredients were also analyzed for AA [method 982.30 E (a, b, c); AOAC Int., 2019] on a Hitachi Amino Acid Analyzer, Model No. L8800 (Hitachi High Technologies America, Inc.; Pleasanton, CA, USA) using ninhydrin for post-column derivatization and nor-leucine as the internal standard.

Monosaccharides in ingredients were analyzed using gas-liquid chromatography based on the individual sugar constituents as alditol acetates after a three-parallel extraction procedure: 1) total non-starch polysaccharides (**NSP**), 2) non-cellulosic polysaccharides (**NCP**), and 3) insoluble non-cellulosic polysaccharides (**I-NCP**). All procedures followed those described by Jaworski et al. (2015). Ingredient samples were also analyzed for stachyose and raffinose, using high-performance liquid chromatography (Dionex App Notes 21 and 92). Total starch was analyzed in ingredients by the amyloglucosidase-alpha-amylase procedure corresponding to the enzymatically hydrolyzed starch converted to glucose, and glucose was quantified by spectrophotometry (method 996.11; AOAC Int., 2019).

### Calculations

Total non-starch polysaccharides in ingredients were calculated using equation 1 (Bach Knudsen, 1997): Total NSP, % = rhamnose + fucose + arabinose + xylose + mannose + galactose + glucose + uronic acids. Cellulose was calculated using equation 2 (Bach Knudsen, 1997): Cellulose, % = (glucose from total NSP extraction)—(glucose from NCP extraction). Insoluble NCP were calculated using equation 3 (Bach Knudsen, 1997): NCP, % = [rhamnose + fucose + arabinose + xylose + mannose + galactose + glucose + (uronic acids from insoluble-NCP)] – cellulose. Soluble NSP were calculated using equation 4 (Bach Knudsen, 1997): soluble-NSP, % = total NSP—cellulose—insoluble-NCP. Values for ATTD of DM, GE, and DE were calculated for each diet for growing pigs, gestating sows, and lactating sows, and the ME of diets fed to growing pigs and gestating sows was also calculated (Adeola, 2001). Using the energy contributions from corn to diets containing corn and SBM or soybean hulls, the ATTD of GE and concentrations of DE in SBM and soybean hulls were calculated by difference (Adeola, 2001). Likewise, the ME in diets fed to growing pigs and gestating sows was also calculated by difference.

### Statistical Analysis

Data were analyzed using the MIXED Procedure (SAS Inst. Inc., Cary, NC, USA). Homogeneity of the variances among treatments was confirmed. Outliers were tested using the UNIVARIATE procedure of SAS, but no outliers were detected. The growing pig, or the gestating or lactating sow was the experimental unit for all analyses. The statistical model included diet or ingredient as the fixed effect and block and replicate within block were random effects. Least square means were calculated, and means were separated using the pdiff option with the Tukey’s adjustment if the model *P*-value was significant. Significance was considered at *P *< 0.05 and a tendency was considered at *P* < 0.10. To compare growing pigs, gestating sows, and lactating sows, data were analyzed using the Mixed procedure of SAS. Within each ingredient, the statistical model included physiological status as the fixed variable. Least square means were calculated, and means were separated using pdiff with the Tukey’s adjustment if the model *P*-value was significant.

## RESULTS

Sows and growing pigs remained healthy during the experiment and feed refusals were not observed. All animals assigned to the experiment completed their feeding periods.

### Growing Pigs

Feed intake by growing pigs fed the corn diet or the SBM diet was less (*P* < 0.05) than by pigs fed the soybean hulls diet, but there was no difference in feed intake between pigs fed the corn diet and the SBM diet (Table 3). The GE intake by pigs fed the soybean hulls diet was greater (*P* < 0.05) than by pigs fed the corn diet, but GE intake of the SBM diet was not different from the other diets. The weight of feces and GE fecal excretion from pigs fed the soybean hulls diet were greater (*P* < 0.05) compared with pigs fed the corn or SBM diets, but there were no differences between the corn and SBM diets. There were no differences in urine weight or GE urine output among the three diets. The ATTD of DM and GE in the soybean hulls diet was less (*P *< 0.05) than in the corn and SBM diets, and the ATTD of GE in the SBM diet was greater (*P* < 0.05) than in the corn diet. The SBM diet had greater DE (*P* < 0.05) than the corn diet, and the soybean hulls diet had less DE (*P* < 0.05) than the corn diet. The SBM diet had greater ME (*P *< 0.05) than the other diets, but there was no difference between the corn diet and the soybean hulls diet. Digestible energy, ME, and DE:GE were less (*P* < 0.05) in soybean hulls than in corn, and corn had less (*P* < 0.05) DE, ME, and DE:GE than SBM. The ME:GE was also less (*P* < 0.05) in soybean hulls than in corn and SBM, but ME:DE was not different among the three ingredients.

**Table 3.** Apparent total tract digestibility (ATTD) of dry matter (DM) and gross energy (GE) and concentrations of digestible (DE) and metabolizable energy (ME) in experimental diets fed to growing pigs^1^, as-fed basis

Item	Corn	Soybean meal	Soybean hulls	SEM	*P*-value
Intake					
Feed, kg/day	1.53^b^	1.61^b^	1.72^a^	0.03	0.001
GE, Mcal/day	5.82^b^	6.20^ab^	6.49^a^	0.11	0.002
Fecal excretion					
Dry feces output, kg/day	0.15^b^	0.14^b^	0.22^a^	0.01	< 0.001
GE, kcal/day	673^b^	602^b^	984^a^	26.9	< 0.001
Urine excretion					
Urine output, kg/day	4.28	4.05	3.61	0.76	0.817
GE, kcal/day	117	168	132	19.2	0.131
ATTD of DM, %	89.4^a^	90.5^a^	85.9^b^	0.34	< 0.001
ATTD of GE, %	88.5^b^	90.3^a^	84.8^c^	0.39	< 0.001
Energy in diets					
DE, kcal/kg	3,356^b^	3,475^a^	3,207^c^	14.9	< 0.001
ME, kcal/kg	3,280^b^	3,371^a^	3,130^b^	22.8	< 0.001
Energy in ingredients^2^					
DE, kcal/kg	3,477^b^	3,827^a^	2,695^c^	43.5	< 0.001
ME, kcal/kg	3,398^b^	3,656^a^	2,608^c^	74.4	< 0.001
DE:GE, %	88^b^	93^a^	70^c^	1.1	< 0.001
ME:DE, %	98	96	97	1.9	0.559
ME:GE, %	86^a^	89^a^	68^b^	1.9	< 0.001

^a-c^Within a row, means without a common superscript differ (*P* < 0.05).

^1^Each least square mean represents 8 observations per diet.

^2^Concentrations of DE and ME in corn were calculated by dividing DE and ME in the corn diet by 96.52%.

### Gestating Sows

No differences in feed intake or GE intake among diets were observed (Table 4), but the weight of dried feces and GE fecal excretion from sows fed the soybean hulls diet were greater (*P* < 0.05) than from sows fed the corn or SBM diets. Weight of urine was not different among the three diets, but GE urine output was greater (*P* < 0.05) from sows fed the SBM diet compared with the other diets, and sows fed the corn diet had less (*P* < 0.05) GE urine output than sows fed the soybean hulls diet. The ATTD of DM and GE in the soybean hulls diet was less (*P *< 0.05) than in the corn and SBM diets, but there was no difference in the ATTD of DM or GE between the corn and SBM diets. Digestible energy was less (*P* < 0.05) in the soybean hulls diet than in the corn diet, and DE was greatest (*P* < 0.05) in the SBM diet. Metabolizable energy was less (*P* < 0.05) in the soybean hulls diet, but there was no difference between the corn and SBM diets. Digestible energy in SBM was greater (*P* < 0.05) than in corn and soybean hulls, but DE was greater (*P* < 0.05) in corn than in soybean hulls. The ME was less (*P* < 0.05) in soybean hulls than in the other ingredients, but there was no difference in the ME between corn and SBM. The DE:GE in soybean hulls was less (*P* < 0.05) than in corn and SBM. The ME:DE and the ME:GE in corn were greater (*P* < 0.05) than in SBM and soybean hulls but the ME:GE was less (*P* < 0.05) in soybean hulls than in SBM.

**Table 4.** Apparent total tract digestibility (ATTD) of dry matter (DM) and gross energy (GE) and concentrations of digestible (DE) and metabolizable energy (ME) in experimental diets fed to gestating sows^1^, as-fed basis

Item	Corn	Soybean meal	Soybean hulls	SEM	*P*-value
Intake					
Feed, kg/day	2.53	2.74	2.70	0.07	0.103
GE, Mcal/day	9.61	10.35	10.23	0.28	0.124
Fecal excretion					
Dry feces output, kg/day	0.23^b^	0.25^b^	0.35^a^	0.01	< 0.001
GE, kcal/day	974^b^	965^b^	1,508^a^	48.7	< 0.001
Urine excretion					
Urine output, kg/day	4.33	6.20	4.33	0.83	0.208
GE, kcal/day	176^c^	396^a^	246^b^	29.4	< 0.001
ATTD of DM, %	89.9^a^	89.9^a^	85.7^b^	0.34	< 0.001
ATTD of GE, %	89.8^a^	90.8^a^	85.2^b^	0.31	< 0.001
Energy in diets					
DE, kcal/kg	3,408^b^	3,497^a^	3,221^c^	11.8	< 0.001
ME, kcal/kg	3,339^a^	3,352^a^	3,129^b^	11.5	< 0.001
Energy in ingredients^2^					
DE, kcal/kg	3,530^b^	3,780^a^	2,559^c^	41.2	< 0.001
ME, kcal/kg	3,459^a^	3,455^a^	2,374^b^	50.7	< 0.001
DE:GE, %	90^a^	92^a^	67^b^	1.05	< 0.001
ME:DE, %	98^a^	91^b^	93^b^	1.08	< 0.001
ME:GE, %	88^a^	84^b^	62^c^	1.32	< 0.001

^a-c^Within a row, means without a common superscript differ (*P* < 0.05).

^1^Each least square mean represents 8 observations per diet.

^2^Concentrations of DE and ME in corn were calculated by dividing DE and ME in the corn diet by 96.52%.

### Lactating Sows

Feed intake and GE intake by lactating sows were not different among diets (Table 5). The ATTD of DM and GE in the corn diet was greater (*P* < 0.05) than in the soybean hulls diet, but the ATTD of DM and GE in the SBM diet was not different from the other diets. The DE in the soybean hulls diet was less (*P* < 0.05) than in the corn diet and the SBM diet, but there was no difference in DE between the corn and SBM diets. The DE and DE:GE of soybean hulls were less (*P* < 0.05) than the other ingredients, but there was no difference between corn and SBM.

**Table 5.** Apparent total tract digestibility (ATTD) of dry matter (DM) and gross energy (GE) and digestible energy (DE) in experimental diets and ingredients fed to lactating sows^1^, as-fed basis

Item	Corn	Soybean meal	Soybean hulls	SEM	*P*-value
Intake					
Feed, kg/day	5.38	5.45	5.03	0.54	0.159
GE, Mcal/day	20.40	20.98	19.00	2.07	0.085
ATTD of DM, %	88.2^a^	87.3^ab^	86.3^b^	0.50	0.048
ATTD of GE, %	87.1^a^	86.8^ab^	85.2^b^	0.62	0.036
DE in diet, kcal/kg	3,303^a^	3,342^a^	3,219^b^	23.9	0.001
DE in ingredients^2^, kcal/kg	3,426^a^	3,545^a^	2,975^b^	71.0	< 0.001
DE:GE in ingredients, %	87^a^	86^a^	78^b^	1.79	0.002

^a-b^Within a row, means without a common superscript differ (*P* < 0.05).

^1^Each least square mean represents 8 observations per diet.

^2^Concentrations of DE and ME in corn were calculated by dividing DE and ME in the corn diet by 96.52%.

### Growing Pigs vs. Gestating Sows and Lactating Sows

The ATTD of DM and GE in the corn diet fed to gestating sows was greater (*P* < 0.05) than if this diet was fed to lactating sows, whereas growing pigs were not different from gestating or lactating sows (Table 6). The DE of the corn diet by gestating sows was greater (*P* < 0.05) than by lactating sows, but growing pigs were not different from gestating sows or lactating sows. The ATTD of GE in corn was greater (*P *< 0.05) by gestating sows than by growing pigs and lactating sows, but the ATTD of GE in corn was not different between growing pigs and lactating sows. Gestating sows had greater (*P *< 0.05) DE for corn than lactating sows, but growing pigs were not different from gestating sows or lactating sows. The ME of the corn diet and of corn fed to gestating sows were also greater (*P* < 0.05) than when fed to growing pigs.

**Table 6.** Apparent total tract digestibility (ATTD) of dry matter (DM) and gross energy (GE) and concentrations of digestible energy (DE) and metabolizable energy (ME) in diets and ingredients fed to growing pigs, gestating sows, and lactating sows^1^, as-fed basis

Item	Growing pigs	Gestating sows	Lactating sows	SEM	*P*-value
Corn					
Energy in diet					
Feed intake, kg/day	1.53^c^	2.53^b^	5.38^a^	0.14	< 0.001
ATTD of DM, %	89.4^ab^	89.9^a^	88.2^b^	0.37	0.009
ATTD of GE, %	88.5^ab^	89.8^a^	87.1^b^	0.41	< 0.001
DE, kcal/kg	3,356^ab^	3,408^a^	3,303^b^	15.5	< 0.001
ME, kcal/kg	3,280^b^	3,339^a^	-	16.5	0.024
Energy in ingredient					
ATTD of GE, %	88.4^b^	89.8^a^	87.1^b^	0.41	< 0.001
DE, kcal/kg	3,477^ab^	3,530^a^	3,426^b^	16.1	< 0.001
ME, kcal/kg	3,398^b^	3,459^a^	-	17.1	0.024
Soybean meal					
Energy in diet					
Feed intake, kg/day	1.61^c^	2.74^b^	5.45^a^	0.16	< 0.001
ATTD of DM, %	90.5^a^	89.9^a^	87.3^b^	0.46	< 0.001
ATTD of GE, %	90.3^a^	90.8^a^	86.8^b^	0.47	< 0.001
DE, kcal/kg	3,475^a^	3,497^a^	3,342^b^	18.0	< 0.001
ME, kcal/kg	3,371	3,352	-	15.2	0.381
Energy in ingredient					
ATTD of GE, %	83.6	86.4	86.3	1.47	0.326
DE, kcal/kg	3,827^a^	3,780^a^	3,545^b^	60.3	0.007
ME, kcal/kg	3,656^a^	3,455^b^	-	50.7	0.014
Soybean hulls					
Energy in diet					
Feed intake, kg/day	1.72^c^	2.70^b^	5.03^a^	0.15	< 0.001
ATTD of DM, %	85.9	85.7	86.3	0.35	0.483
ATTD of GE, %	84.8	85.2	85.2	0.36	0.745
DE, kcal/kg	3,207	3,221	3,219	13.8	0.745
ME, kcal/kg	3,130	3,129	-	15.7	0.997
Energy in ingredient					
ATTD of GE, %	70.3^b^	67.0^b^	77.7^a^	1.80	0.001
DE, kcal/kg	2,695^b^	2,559^b^	2,975^a^	68.9	0.001
ME, kcal/kg	2,608	2,374	-	78.6	0.054

^a-c^Within a row, means without a common superscript differ (*P* < 0.05).

^1^Each least square mean represents 8 observations per diet or ingredient.

The DE and the ATTD of DM and GE by lactating sows of the SBM diet were less (*P* < 0.05) than by growing pigs or gestating sows, but there was no difference between growing pigs and gestating sows. The ME in the SBM diet was also not different between growing pigs and gestating sows and no differences in ATTD of GE of SBM between growing pigs, gestating sows, and lactating sows were observed. There was no difference in DE of SBM between growing pigs and gestating sows, but DE of SBM by lactating sows was less (*P* < 0.05) than by growing pigs or gestating sows. The ME of SBM by growing pigs was greater (*P* < 0.05) than by gestating sows.

The DE and the ATTD of DM and GE in the soybean hulls diet were not different among growing pigs, gestating sows, and lactating sows, but the DE and ATTD of GE in soybean hulls fed to lactating sows were greater (*P* < 0.05) than when fed to growing pigs or gestating sows.

## DISCUSSION

Although the concentration of GE in corn was close to the expected value, crude protein in corn was less than reported (NRC, 2012). Likewise, GE in SBM and soybean hulls was less than reported (NRC, 2012; Rodriguez et al., 2020), but crude protein and acid hydrolyzed ether extract in SBM agreed with current book values (NRC, 2012). The analyzed fat, crude protein, and TDF in SBM and soybean hulls were also in agreement with NRC (2012), and analyzed concentrations of amino acids in the three ingredients were in agreement with expected values.

The observation that the main monosaccharides in the non-cellulosic part of the corn fiber was xylose and arabinose is in agreement with data from Jaworski et al. (2015) and reflects that the main part of corn fiber consists of arabinoxylans. Likewise, the concentration of cellulose in corn fiber agreed with Jaworski et al. (2015), and there appears to be about twice as much arabinoxylan as cellulose in corn fiber. The high concentrations of galactose and arabinose in the insoluble fiber from SBM indicates a high concentration of the pectic polysaccharides arabinogalactans and rhamnogalacturonans in SBM fiber (Navarro et al., 2019), although the low concentration of rhamnose indicates that there likely are more arabinogalactans than rhamnogalacturonans in fiber from SBM. The observation that the concentration of monosaccharides in the fiber from soybean hulls appears to be different from the monosaccharides in SBM indicates that the fiber composition of the hulls is different from the composition of fiber in other parts of the soybean. Based on the monosaccharide composition in the fiber in soybean hulls, it is speculated that the non-cellulosic fibers in soybean hulls primarily consist of xylogalacturonan and arabinans and possibly some arabino galactans and rhamnogalacturonans. However, whereas the insoluble fiber in corn and soybean meal contains only around 25% cellulose, the insoluble fiber in soybean hulls contain more than 60% cellulose. These differences in composition of the insoluble fiber may result in different fermentation characteristics in pigs, but because fermentation characteristics were not determined in this experiment, this hypothesis cannot be confirmed from the current data.

Growing pigs were provided feed at three times the maintenance requirements for ME, which is close to ad libitum intake for growing pigs, whereas gestating sows were fed 1.5 times the maintenance requirement for ME, which is close to recommended levels for commercially fed sows (NRC, 2012). Likewise, lactating sows were offered ad libitum access to feed as is common under commercial conditions. Thus, the DE and ME in all ingredients obtained in this work are likely to be close to what will be obtained under commercial conditions.

All diets had analyzed GE that agreed with calculated values, which indicates that errors in diet mixing, subsampling, or GE analysis were minimized. The ATTD of GE, and DE and ME in corn fed to growing pigs agreed with established values (NRC, 2012). However, ATTD of GE and DE and ME in SBM were greater than reported by NRC (2012), but in agreement with other recent values (Wang et al., 2022; Kim et al., 2024). The greater DE in SBM fed to growing pigs compared with NRC (2012) is also in agreement with recent data (Sotak-Peper et al., 2015) and demonstrates that current genetics of pigs appear to be able to better digest energy in the sources of SBM that are now being used than older genotypes. The greater ME in SBM fed to growing pigs compared with NRC (2012) is a result of the greater ME to DE ratio in SBM. Because the difference between DE and ME is the energy excreted in the urine, which primarily consists of nitrogen from deaminated amino acids, it appears that pigs used in this experiment and in some other recent experiments have greater retention of nitrogen and reduced excretion of nitrogen in the urine, than pigs used in earlier experiments have. These observations, therefore, indicate that dietary nitrogen was utilized more efficiently in the pigs than in previous experiments, which is consistent with recent data that demonstrated that growing pigs of modern genotypes are more efficient at retaining nitrogen compared with pigs of older genotypes (Millet et al., 2018).

The greater DE and ME in soybean hulls fed to growing pigs compared with some previous values is a result of the greater ATTD of GE compared with the values from NRC (2012) and Rodgriguez et al. (2020), although concentrations of fiber were not different among the sources. The ATTD of GE, DE, and ME in soybean hulls by gestating sows were in agreement with recent data (Wang et al., 2022).

Because gestating sows can ferment more nutrients than growing pigs, diets are sometimes believed to have greater energy values when fed to gestating sows compared with growing pigs (Le Goff and Noblet, 2001; Casas and Stein, 2017). Although ME in corn was greater in gestating sows than in growing pigs in this experiment, this was not the case for ME in SBM and soybean hulls, which confirms that sows do not always have greater energy digestibility than growing pigs as has been previously reported (Lowel et al., 2015). Likewise, the digestibility of energy in mixed diets is not always greater in gestating sows than in growing pigs (Shipman et al., 2023). The greater ME in SBM fed to growing pigs versus gestating sows indicates that growing pigs retain nitrogen with greater efficiency than gestating sows, which was also reflected in the greater ME:DE ratio. It is likely that gestating sows had reduced protein requirement compared with growing pigs and diets containing SBM, therefore, contained amino acids above the requirements for gestating sows, which may have resulted in reduced protein utilization compared with growing pigs. Protein efficiency, therefore, may have been reduced in gestating sows compared with growing pigs, which may be the reason for the reduced ME to DE ratio (Lowel et al., 2015).

The observation that ATTD of GE and DE in diets containing corn and SBM were greater in gestating sows than in lactating sows is likely because gestating sows were fed at approximately 1.5 times the maintenance requirements for energy, whereas lactating sows were allowed ad libitum intake of diets. Greater feed intake results in a greater passage rate, which reduces the digestibility of DM and energy (Cunningham et al., 1962; Shi and Noblet 1993; Le Goff and Noblet 2001; Kim et al., 2007). Therefore, the ATTD of GE in corn and SBM fed to lactating sows was less than in gestating sows. However, these values likely reflect the ATTD of diets used in commercial settings, because the levels of feed intake used in this experiment are close to commercially used levels. The reason for the greater DE of soybean hulls in lactating sows compared with growing pigs is likely a result of the longer hindgut in sows, which results in more microbes that can ferment the fiber in soybean hulls. The observation that DE of soybean hulls was also greater in lactating sows than in gestating sows seems counterintuitive because feed intake in lactating sows is greater than in gestating sows, which might have been assumed to result in reduced digestibility of nutrients. However, in a previous experiment, DE in a corn-SBM diet was not different between gestating and lactating sows, but DE in defatted-rice bran, which is also a high-fiber ingredient, was greater when fed to lactating sows than to gestating sows (Casas et al., 2022). It is, therefore, possible that lactating sows have a greater ability to ferment dietary fiber than gestating sows, which may be related to increased microbial activity in lactating sows, but we are not aware of data determining microbial activity in lactating sows so we can only speculate on this mechanism, but this is an area that deserves future attention. Nevertheless, the observation that DE in soybean hulls fed to lactating sows was almost 3,000 kcal/kg indicates that it may be possible to use soybean hulls in diets for lactating sows without reducing DE in the diets. Because of the greater digestibility of GE in soybean hulls that were observed in this experiment, future research is needed to determine inclusion rates of soybean hulls in diets for gestating and lactating sows.

One potential limitation of the current work is that the total collection procedure was used to calculate digestibility in both growing pigs and gestating sows, whereas the grab sampling technique was used for the lactating sows with a subsequent calculation of digestibility based on titanium concentrations in diets and fecal samples. This approach was used because it is not possible to place lactating sows in metabolism crates. However, if the titanium procedure and the total tract procedure do not result in the same values for digestibility, this could potentially affect results. Comparisons in growing pigs of results obtained with the titanium procedure, or another indigestible marker, and the total collection procedure did not result in clear differences between the two procedures (Li et al., 2016; Huang et al., 2018). However, we are not aware of any such comparisons in sows but assume that there also are no differences between the two procedures in sows.

## CONCLUSION

The hypothesis that energy digestibility of diets fed to gestating or lactating sows is always greater than in growing pigs was not confirmed because differences in digestibility of energy and concentrations of energy among gestating sows, lactating sows, and growing pigs depended on the feed ingredients used in the diets. However, it was concluded that in accordance with the hypothesis, sows had greater utilization of energy from soybean hulls than current book values but research is needed to determine optimum inclusion levels in diets for sows. It was also confirmed that when fed to growing pigs, SBM contains more DE and ME than previously accepted values.
